# In vitro assessment of an antimicrobial peptide against *Acinetobacter baumannii* persister cells

**DOI:** 10.1038/s41598-025-33137-w

**Published:** 2025-12-26

**Authors:** Mandana Hosseini, Farzaneh Hosseini, Nour Amirmozafari, Abbas Akhavan Sepahy

**Affiliations:** 1 Department of Microbiology, NT.C, Islamic Azad University, Tehran, Iran; 2 Department of Microbiology, School of Medicine, Iran University of Medical Sciences, Tehran, Iran

**Keywords:** *Acinetobacter baumannii*, Antimicrobial peptide, Multidrug resistance, Gene expression, Biotechnology, Microbiology

## Abstract

The rise of multidrug-resistant and persister cell populations of *Acinetobacter baumannii* (*A. baumannii*) poses a significant threat in healthcare settings, highlighting the need for novel therapeutic strategies. This study investigates a specifically designed antimicrobial peptide and its potential activity against this pathogen. Using advanced bioinformatics, a 20-amino acid antimicrobial peptide was designed and synthesized. The peptide’s efficacy was evaluated in vitro through MIC assays against *A. baumannii*, along with assessments of its effects on persister cells, biofilm formation, and gene expression (pmrB and lasI) using quantitative PCR. The designed peptide exhibited a potent MIC value of 64 µg/mL, reducing persister cell populations of *A. baumannii* by 75% within 24 h (p < 0.0001). It significantly inhibited biofilm formation, with OD reductions of up to 5.4 log at 64 µg/mL (p < 0.0001). Real-time PCR analysis revealed a 6.2-fold upregulation of pmrB and 3.7-fold upregulation of lasI after 24 h (p < 0.0001), indicating bacterial adaptive responses. The antimicrobial peptide demonstrated strong antibacterial and antibiofilm activity against *A. baumannii*, though with moderate cytotoxicity (8–17% reduction in cell viability). These findings suggest a promising avenue for developing novel antimicrobial strategies.

## Introduction

*Acinetobacter baumannii* has emerged as a major challenge in modern healthcare due to its ability to cause persistent and hard-to-treat nosocomial infections, particularly in immunocompromised patients and individuals undergoing invasive procedures. Known for its remarkable capacity to survive in hospital environments and tolerate antimicrobial treatments, this pathogen is responsible for infections such as pneumonia, bloodstream and wound infections, contributing substantially to patient morbidity and mortality^1,2^.

The pathogenic success of *A. baumannii* is largely attributed to its remarkable ability to survive under antibiotic pressure and adapt to hostile environments, rather than solely to multidrug resistance^3,4^. This adaptability is facilitated by several mechanisms, including the acquisition of resistance determinants, modification of antimicrobial targets, and, most importantly, the formation of biofilms^5,6^. Biofilms act as protective barriers that enhance bacterial survival by shielding cells from immune responses and antimicrobial agents^7^. Within these biofilms, *A. baumannii* also harbors persister cells, which are dormant phenotypic variants capable of withstanding otherwise lethal antibiotic concentrations. These cells play a critical role in treatment failure and infection recurrence, representing one of the major challenges in the clinical management of *A. baumannii* infections^8,9^.

Recent developments have directed attention toward alternative strategies that specifically target biofilm-embedded and persister cells. Antimicrobial peptides (AMPs) have emerged as promising candidates due to their broad-spectrum antibacterial activity and their ability to disrupt bacterial membranes, ultimately leading to cell death. AMPs are short peptides, typically ranging from 10 to 100 amino acids in length, and constitute a key component of the innate immune defense across diverse organisms^10^. Their unique potential to penetrate biofilms and eliminate tolerant or dormant bacterial subpopulations provides a novel therapeutic approach to overcoming the limitations of conventional treatments against *A. baumannii* infections^11,12^.

The efficacy of AMPs against *A. baumannii* is influenced by key genetic factors, particularly the *pmrB* and *lasI* genes. The *pmrB* gene, part of the PmrAB two-component system, is crucial for regulating membrane modifications that confer resistance to cationic AMPs, facilitating bacterial survival in hostile environments^13^. The *lasI* gene is a key component of quorum sensing and biofilm formation, which are critical for persistence and chronic infection^14^. Understanding and targeting these genes provides insight into the adaptive mechanisms of *A. baumannii*, informing potential strategies to combat infections associated with this pathogen.

This study focuses on the design and evaluation of a novel AMP tailored to combat *A. baumannii*. The research investigates the peptide’s antibacterial activity, its ability to inhibit biofilm formation, and its efficacy against persister cell populations in a controlled in vitro setting. Additionally, the study examines changes in gene expression associated with bacterial adaptive responses, providing insights into the mechanisms employed by *A. baumannii* in response to AMP exposure.

Given the critical need for alternative treatments against *A. baumannii*, this study is of significant importance. The reference strain *A. baumannii* ATCC 17,978 serves as a valuable model for investigating the behavior of persister cells, which pose a major challenge in chronic infections. We evaluated a short linear 20-amino-acid AMP with optimized amphipathic and cationic properties. This peptide was comprehensively evaluated for its antibacterial, anti-persister efficacy, and impact on gene expression (*pmrB* and *lasI*) in *A. baumannii*. By exploring the potential of AMPs, this research highlights the importance of targeting persister cells to overcome their contribution to infection persistence, ultimately providing insights for the development of novel antimicrobial strategies. Understanding bacterial adaptive responses to these peptides may further inform future approaches to improve treatment outcomes.

## Results

### Peptide and primer design evaluation

The designed AMP, composed of a linear sequence of 20 amino acids (RRFFKKAAHVGKHVGKAARR), was structurally characterized to evaluate features relevant to bacterial membrane interaction. The peptide exhibited a net positive charge of + 8.5 and a theoretical isoelectric point (pI) of 12.48, confirming its cationic nature and strong electrostatic potential toward negatively charged bacterial surfaces. Its balanced hydrophobic composition (~ 40%) supports efficient membrane insertion and disruption.

**Fig. 1:**
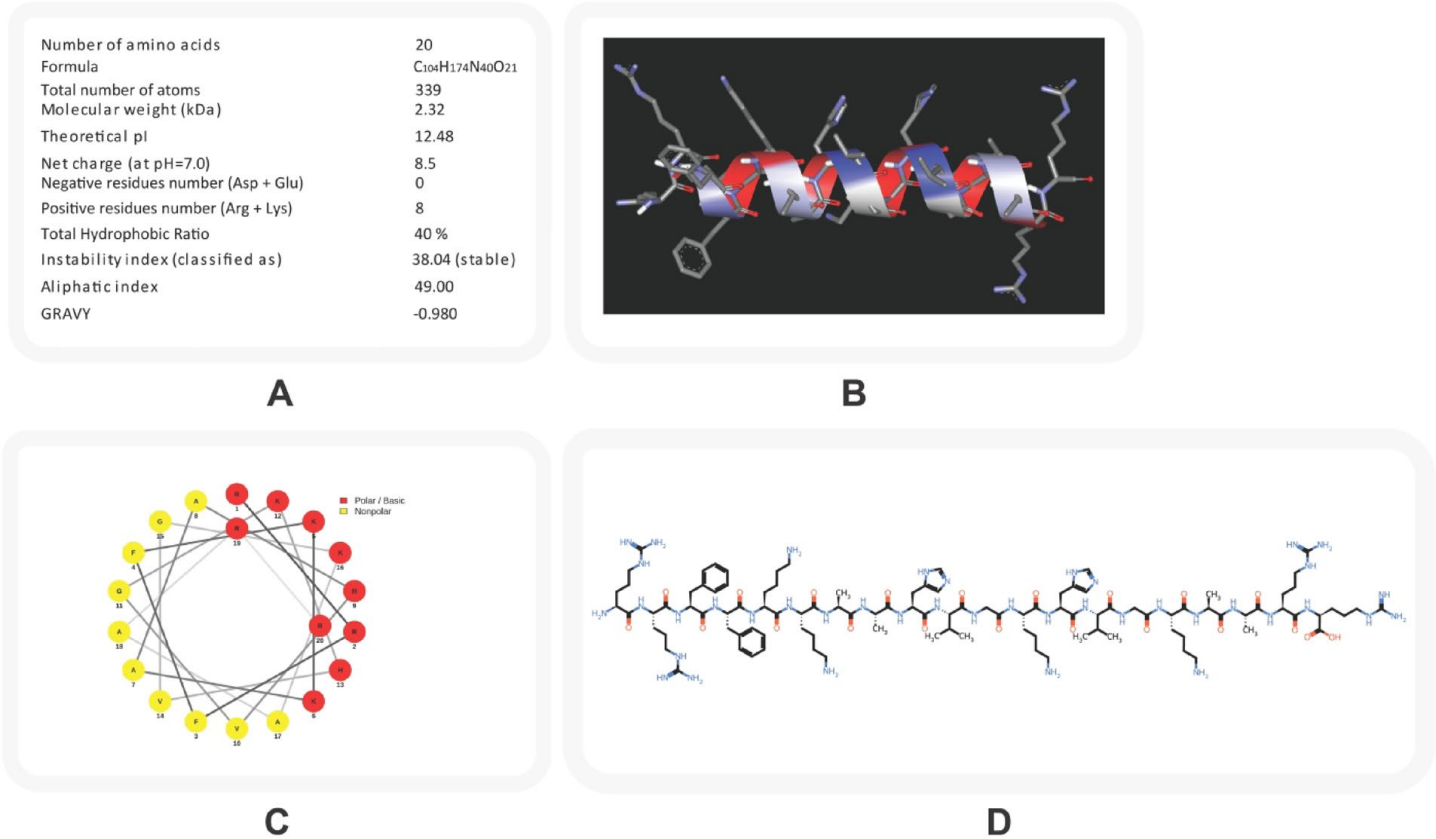
Structural and physicochemical characterization of the designed AMP.**A** Physicochemical properties predicted using the ProtParam tool, indicating several features of the peptide. **B** Three-dimensional structural model of the peptide generated by PEP-FOLD4, showing hydrophilic (blue) and hydrophobic (red) residues. **C** Helical wheel projection illustrating the amphipathic nature of the peptide; red circles denote polar residues and yellow circles represent nonpolar residues. **D** SMILES-based chemical structure of the peptide generated using the PepSMI tool.

The physicochemical and structural characteristics of the peptide are illustrated in Fig. 1. Panel (A) summarizes the physicochemical parameters calculated using ProtParam, including molecular weight (2.32 kDa), hydrophobicity ratio (40%), and instability index (38.04), indicating structural stability. Panel (B) presents the 3D structural model highlighting hydrophilic (blue) and hydrophobic (red) residues. Panel (C) displays the helical wheel diagram, showing an amphipathic arrangement of polar and nonpolar residues, while panel (D) depicts the SMILES-based chemical structure of the peptide (Fig. 1).

### Antibacterial activity of the designed peptide

**Fig. 2:**
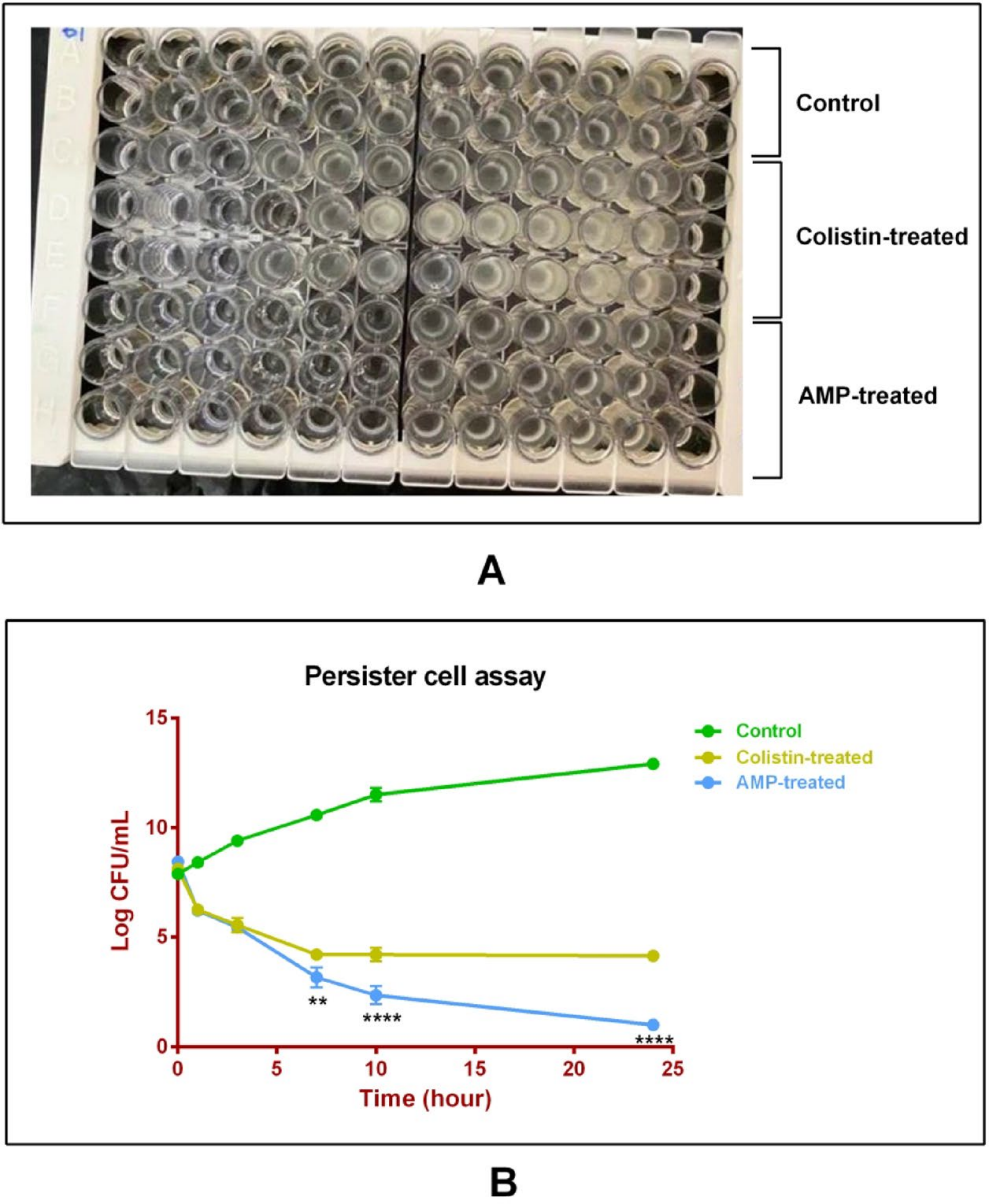
Antibacterial and anti-persister activity of the designed AMP. **A** Broth microdilution assay illustrating the antibacterial activity of the designed antimicrobial peptide and colistin against *A. baumannii* ATCC 17,978. The top two rows represent the growth control wells (no antimicrobial treatment). The next three rows correspond to serial twofold dilutions of colistin, and the three rows below correspond to serial twofold dilutions of the designed peptide. Wells showing no visible turbidity indicate inhibition of bacterial growth at the respective concentration. **B** Growth curves of control (no antimicrobial treatment), peptide-treated, and colistin-treated samples over 24 h. Statistical significance vs. control was determined by Tukey’s multiple comparisons test: ** *p* < 0.01, **** *p* < 0.0001. Data are presented as mean ± standard deviation (SD) of three independent experiments (*n* = 3).

The antibacterial potential of the designed peptide was evaluated against *A. baumannii* ATCC 17,978 using the broth microdilution method. The peptide exhibited an MIC of 64 µg/mL and an MBC of 256 µg/mL, demonstrating substantial antibacterial activity. For comparison, colistin, used as a clinical reference, showed an MIC of 1 µg/mL, consistent with its established potency as a last-resort antibiotic. Growth inhibition was quantified by measuring optical density at 600 nm (OD₆₀₀), as shown in Fig. 2 (panel A).

### Persister cell formation

**Fig. 3:**
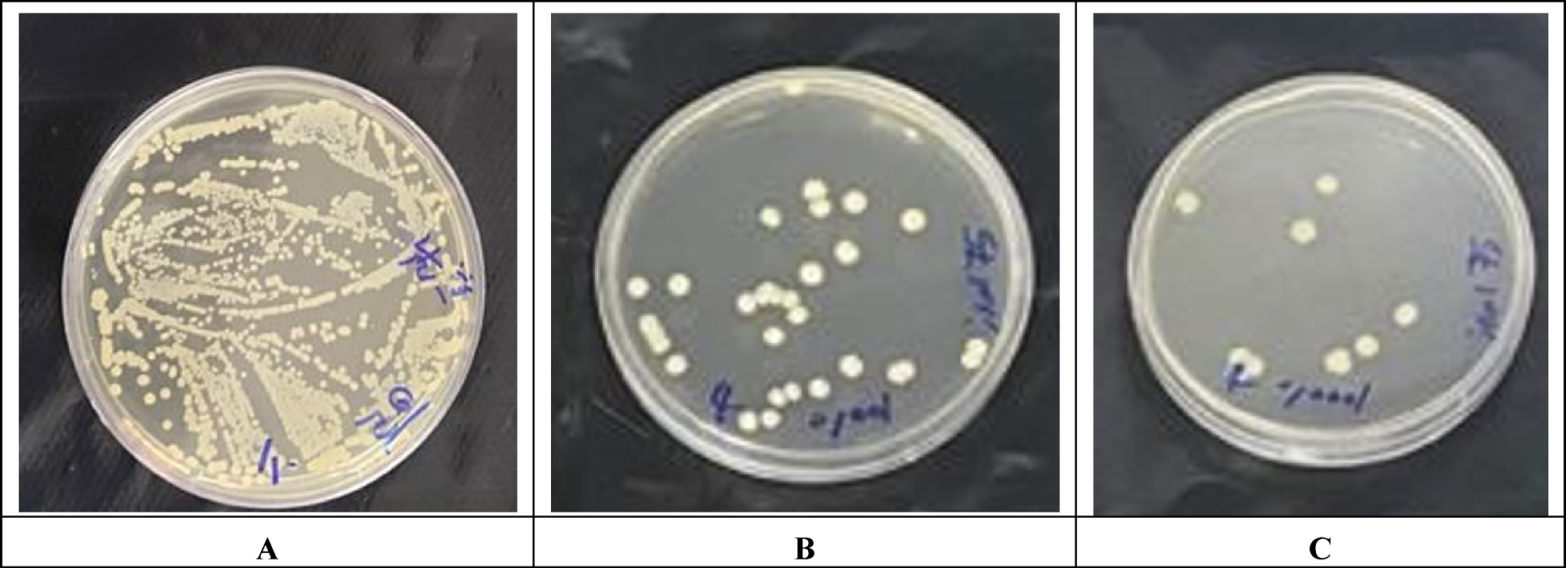
Representative agar plates showing colony growth of *A. baumannii* under different treatment conditions. Bacterial survival was evaluated following exposure to the designed AMP and colistin. (A) the untreated control showing dense colony growth, (B) cultures treated with colistin, and (C) cultures treated with the designed peptide. A marked reduction in colony numbers was observed in treated samples compared with the control.

Persister cell formation in *A. baumannii* was induced by exposure to colistin at tenfold its MIC (10 µg/mL). After 7 h of treatment, a stable population of persister cells was detected, as illustrated in Fig. 2 (panel B). Subsequent treatment with the designed peptide (64 µg/mL) markedly reduced this population, resulting in approximately 75% reduction after 24 h (Fig. 2). Statistical analysis confirmed the significance of this reduction (*p* < 0.0001), highlighting the peptide’s strong anti-persister activity. The statistical evaluation of peptide-mediated effects on persister cell survival is summarized in Table 2. The agar plate containing colonies of *A. baumannii* under various conditions is shown in Fig. 3. The findings suggest that the designed peptide effectively targets metabolically dormant *A. baumannii* cells, which are typically tolerant to conventional antibiotics and associated with chronic or recurrent infections.

**Table 2:** The statistical outcomes derived from examining the effects of the investigated peptide on persister cell subpopulations.

Tukey’s multiple comparisons test	Mean diff.	95.00% CI of diff.	Significant?	Adjusted *P* value
0 h	0.000	-0.7748 to 0.7748	No	> 0.9999
1 h	0.000	-0.7748 to 0.7748	No	> 0.9999
3 h	0.000	-0.7748 to 0.7748	No	> 0.9999
7 h	1.067	0.2919 to 1.841	Yes	0.0036
10 h	1.800	1.025 to 2.575	Yes	< 0.0001
24 h	3.133	2.359 to 3.908	Yes	< 0.0001

### Antibacterial efficacy in *A. baumannii*-infected HT29 cellsctivity of the designed peptide 

**Fig. 4:**
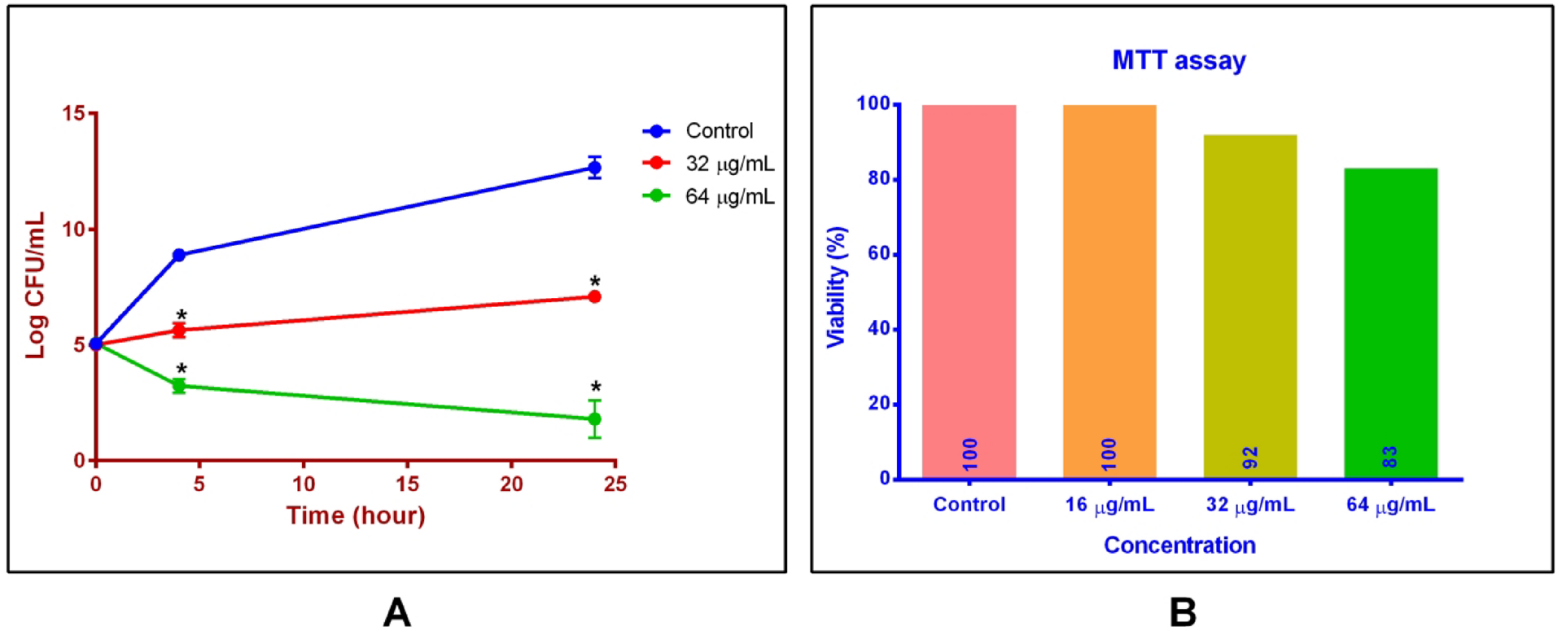
Antibacterial activity and cytotoxicity assessment in HT29 epithelial cell cultures. **A** Colony enumeration of *A. baumannii* after treatment with the designed peptide at 32 and 64 µg/mL compared with untreated control samples, demonstrating dose-dependent antibacterial effects in infected HT29 cells. Statistical significance vs. control was determined by Tukey’s multiple comparisons test: * *p* < 0.05. **B** Cytotoxicity profile of the peptide on HT29 cells at the same concentrations, showing the percentage of viable cells and indicating a tolerable cytotoxicity range.

The antibacterial activity of the designed peptide was evaluated in *A. baumannii*-infected human HT29 epithelial cells. As shown in Fig. 4, treatment with 32 µg/mL of the peptide reduced intracellular bacterial viability by 3.23 and 5.4 log CFU after 4 and 24 h, respectively. A higher concentration (64 µg/mL) achieved greater reductions of 5.6 and 10.7 log CFU at the same time points (*p* < 0.0001). These findings demonstrate the peptide’s strong antibacterial efficacy against intracellular *A. baumannii*.

### Cytotoxicity assessment

The cytotoxicity of the peptide toward HT29 cells was evaluated using the MTT assay. As illustrated in Fig. 4, treatment with 32 and 64 µg/mL of the peptide reduced cell viability by only 8% and 17%, respectively, compared with untreated controls, indicating a favorable cytotoxicity profile at bactericidal concentrations. Additionally, *pho*P expression remained unchanged under these conditions, suggesting minimal activation of host cellular stress pathways.

### Anti-biofilm activity assessment

**Fig. 5:**
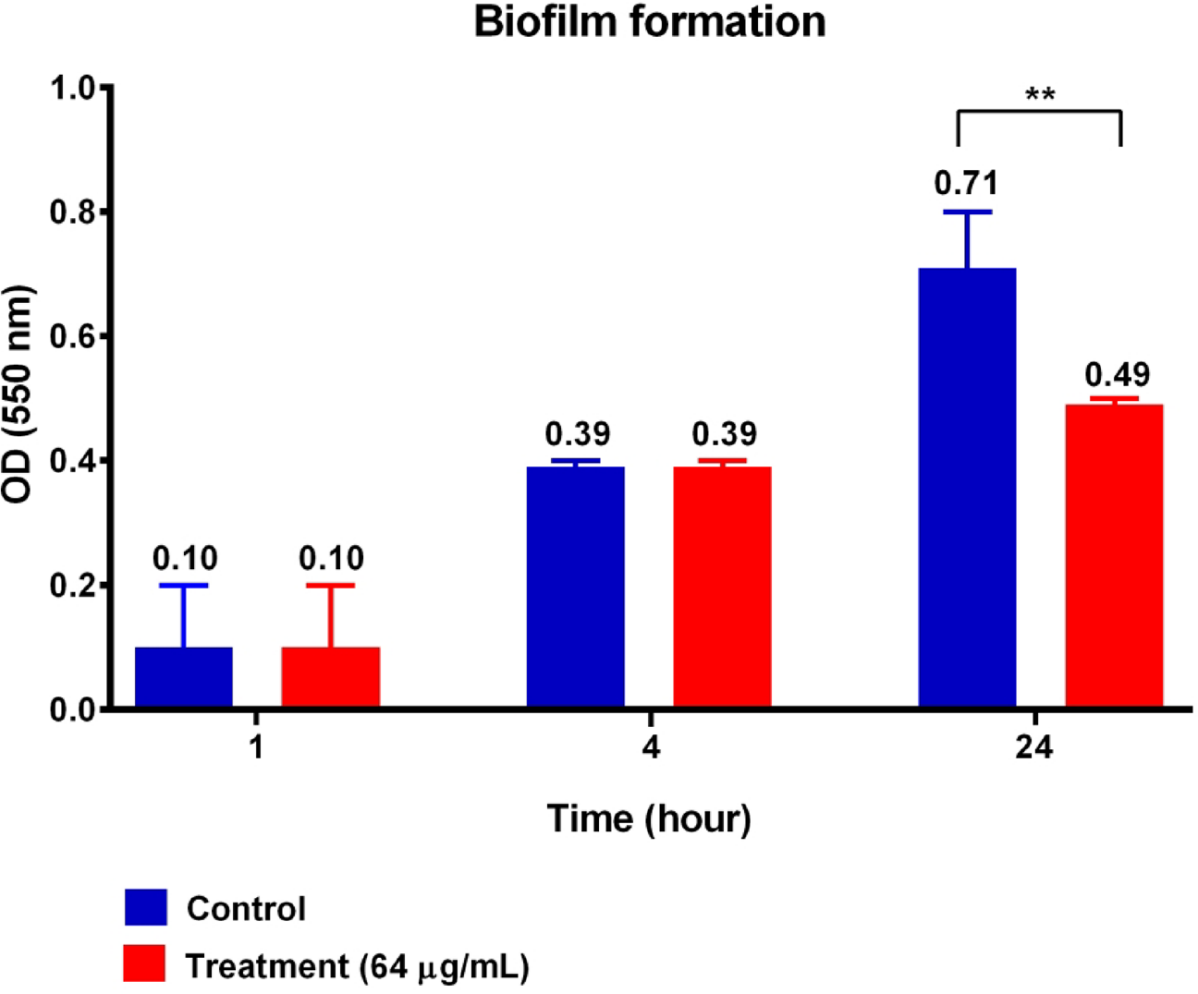
Time-dependent inhibitory effect of the designed AMP on *A. baumannii* biofilm formation. No significant reduction in biofilm formation was observed at early time points (1 h and 4 h). However, treatment with the peptide (64 µg/mL) significantly reduced biofilm biomass after 24 h, as indicated by a decrease in mean OD_550_ from 0.71 to 0.49 (*p* = 0.0056).

No significant reduction in *A. baumannii* biofilm formation was observed at early time points (1 h and 4 h). However, treatment with the designed AMP (64 µg/mL) significantly reduced *A. baumannii* biofilm formation after 24 h. The mean OD₅₇₀ value decreased from 0.71 to 0.49 over time (*p* = 0.0056). These results indicate that the peptide exerts a time-dependent inhibitory effect on biofilm development (Fig. 5).

### Gene expression analysis

**Fig. 6:**
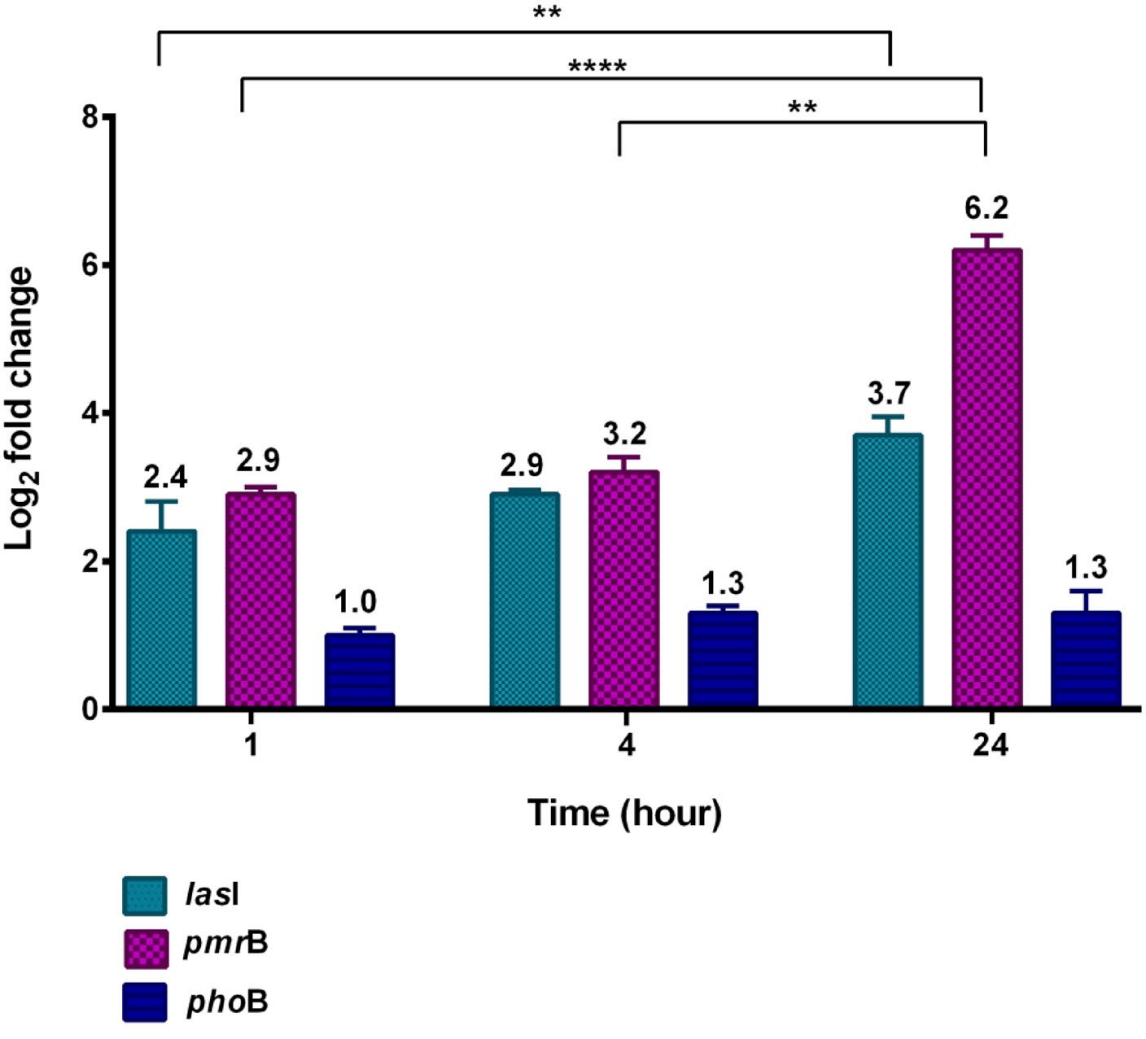
Expression of target genes in *A. baumannii* following peptide treatment. Relative expression levels of *pmr*B and *las*I were measured at 1, 4, and 24 h after exposure to the designed peptide, compared with untreated control samples. 16 S rRNA was used as the internal reference for normalization, and fold changes are presented to illustrate the peptide-induced stress response. Statistical significance was determined by Tukey’s multiple comparisons test: ** *p* < 0.01, **** *p* < 0.0001.

To elucidate the molecular response of *A. baumannii* to the designed peptide, the expression levels of two key genes (*pmr*B, associated with antibiotic resistance, and *las*I, involved in quorum-sensing regulation) were quantified by real-time PCR (Fig. 6). Following treatment with 32 µg/mL of the peptide, a significant upregulation was observed for both genes compared to the untreated control (*p* < 0.0001). Specifically, *pmr*B expression increased 3.2-fold after 4 h and 6.2-fold after 24 h, while *las*I expression rose 2.9-fold and 3.7-fold at the corresponding time points. Expression data were normalized against 16 S rRNA as the internal reference. These findings indicate that the peptide induces a stress-adaptive response in *A. baumannii*, potentially influencing both resistance and quorum-sensing pathways.

## Discussion

The emergence of multidrug-resistant *A. baumannii* as a major nosocomial pathogen poses a significant challenge to healthcare systems worldwide. Renowned for its ability to survive under harsh conditions and resist multiple antibiotics, *A. baumannii* is a leading cause of hospital-acquired infections, particularly in intensive care units^1^. The increasing prevalence of carbapenem-resistant strains underscores the urgent need for alternative therapeutic strategies. In this context, AMPs offer a promising approach due to their unique mechanisms of action, which can bypass conventional resistance pathways and target challenging bacterial populations, such as persister cells.

AMPs are naturally occurring molecules that play a vital role in the innate immune response across diverse organisms. They exhibit broad-spectrum antibacterial activity, targeting both Gram-positive and Gram-negative bacteria. Their primary mechanism of action involves disruption of bacterial cell membranes, ultimately causing cell lysis. This distinctive mode of action is especially advantageous against resistant strains, as it lowers the likelihood of resistance development compared to conventional antibiotics^10^. Moreover, AMPs’ membrane-targeting properties make them promising candidates for eliminating challenging bacterial populations, including persister cells, which are highly tolerant to traditional antimicrobial therapies.

Recent research has focused on the design and optimization of synthetic AMPs to enhance their efficacy against specific pathogens, including *A. baumannii*. Several recent studies have evaluated AMPs as potential agents against multidrug-resistant *A. baumannii*, with important implications for persister and biofilm control. Notably, SAAP-148 and a number of synthetic membrane-active peptides have been reported to eradicate persister cells and disrupt biofilms, demonstrating that membrane-targeting AMPs can overcome tolerance mechanisms that limit conventional antibiotics^15^. In *A. baumannii* specifically, designed peptides such as ZY4 have shown potent antibacterial and antibiofilm activity against clinical and reference strains, supporting the feasibility of AMP-based interventions for this pathogen^16^. Another study also report very low MICs for some peptides (for example, Cec4 with MICs in the low µg·mL⁻¹ range against many *A. baumannii* isolates), illustrating the range of potencies achievable by different sequences and structural classes^17^. Our study complements this prior art by testing a rationally designed, short, linear α-helical cationic AMP. In the present study, the newly developed peptide demonstrated significant antibacterial activity, with an MIC of 64 µg/mL. Although this MIC is higher than colistin’s 1 µg/mL, it reflects a distinct mechanism of action targeting resistant strains. The peptide in the present study effectively reduced bacterial growth, illustrating the potential benefits of rational peptide design for targeting specific pathogens.

The peptide designed in this study belongs to the class of short, linear, synthetic cationic antimicrobial peptides. Its amino-acid composition and helical wheel projection indicate that it adopts an amphipathic α-helical conformation, a common structural motif among membrane-active AMPs. Unlike naturally occurring peptide families (e.g., defensins, cathelicidins, or cyclic peptides), this AMP is rationally designed in silico to optimize physicochemical parameters such as net positive charge, hydrophobicity, and helix-forming potential. Such peptides are classified under α-helical AMPs with membrane-disruptive activity, a well-established group known for potent broad-spectrum antimicrobial effects.

One of the major challenges in treating *A. baumannii* infections is its capacity to form biofilms^18^, structured bacterial communities embedded in a protective extracellular matrix that enables evasion of immune responses and antibiotic treatment. The present study demonstrates that the designed AMP significantly inhibits biofilm formation after 24 h, suggesting it may enhance the efficacy of conventional antibiotics. This finding aligns with previous reports showing that AMPs can disrupt biofilm integrity, thereby improving therapeutic outcomes.

The log reductions in bacterial viability observed at 32 µg/mL (3.23 and 5.4) and 64 µg/mL (5.6 and 10.7, *p* < 0.0001) further highlight the peptide’s potency. These results surpass the antibacterial performance of many natural AMPs, such as Pleurocidin (25 amino acids)^19^, which exhibit limited efficacy against multidrug-resistant biofilms^20^. The peptide’s strong cationic nature (net charge 8.5, pI 12.48) and the strategic arrangement of arginine, lysine, and hydrophobic residues enhance its ability to penetrate bacterial membranes, a design feature aimed at overcoming *A. baumannii’s* resistance mechanisms. This potent antibacterial activity is complemented by a significant reduction in biofilm formation, with a 5.4 log OD decrease (*p* < 0.0001), consistent with prior studies showing that AMPs can disrupt the extracellular matrix of bacterial biofilms^21,22^. Together, these findings support the dual functionality of the peptide in targeting both planktonic bacteria and biofilm-associated cells, reinforcing its potential as a therapeutic candidate.

The impact of the AMP on persister cell populations is another noteworthy aspect of this study. Persister cells are a small subpopulation of bacteria that enter a dormant state, rendering them highly tolerant to antibiotics. The peptide achieved a remarkable 75% reduction in persister cell populations within 24 h (*p* < 0.0001). These dormant cells are often responsible for infection recurrence and chronic disease states^8^, highlighting that targeting them represents a critical advancement in the fight against antibiotic resistance.

Gene expression analysis further elucidates the interaction between the AMP and *A. baumannii* (Fig. 5). Treatment with the peptide induced significant upregulation of resistance-related genes, including *pmr*B (6.2-fold) and *las*I (3.7-fold) at 24 h (*p* < 0.0001), reflecting *A. baumannii*’s adaptive response, supported by recent genomic analysis^23^, suggesting targeted inhibition of these pathways in future designs. The upregulation of *pmr*B, a component of the PmrAB two-component system, indicates activation of a key resistance mechanism against cationic AMPs. PmrAB mediates the addition of phosphoethanolamine (pEtN) to lipid A in the lipooligosaccharide (LOS), reducing membrane negative charge and hindering peptide binding^24^, consistent with the peptide’s cationic nature (net charge + 8.5, pI 12.48). The 6.2-fold increase at 24 h is consistent with recent findings by Ko SY et al.^25^, which demonstrated that *Pmr*AB activation under acidic conditions (pH 5.5) can increase colistin resistance by up to 32-fold, reflecting a similar stress-induced adaptation. Notably, *pho*P expression remained unchanged, suggesting selective activation of resistance pathways, likely because PhoPQ responds primarily to environmental cues rather than peptide exposure. Another study by Xie et al.^23^ used genomic sequencing to show that *Pmr*AB mutations are frequent in MDR A. *baumannii* isolates, reinforcing its role in peptide resistance, while *Pho*PQ mutations were less common, supporting our observation of no *pho*P change. These findings align with Charretier et al.^26^, who highlighted *Pmr*AB’s role in peptide resistance, and Ko SY et al.^25^, confirming its activation under stress, while Bhagirath et al.^27^ and Yamada et al.^13^ reviewed studies that support the minimal role of *Pho*PQ in this context.

The 3.7-fold upregulation of *las*I at 24 h suggests activation of quorum-sensing pathways, potentially coordinating biofilm formation and virulence. Although *las*I is classically studied in *P. aeruginosa*, *A. baumannii* harbors analogous systems (AbaI/AbaR) regulating similar pathogenic traits^28^. Importantly, despite this upregulation, the AMP demonstrated potent antibacterial activity, achieving a 75% reduction in persister cell populations (*p* < 0.0001) and a 5.4-log OD decrease in biofilms at 64 µg/mL.

This distinction is critical: persister cells represent a dormant subpopulation that can arise independently in planktonic cultures or within biofilms. In this study, persister cells were derived from planktonic cultures, as described in the Methods. Therefore, the observed reductions reflect eradication of planktonic persisters rather than direct biofilm disruption, underscoring the AMP’s capacity to target highly tolerant bacterial populations while also limiting biofilm biomass.

The cytotoxicity evaluation revealed minimal toxicity, with only 8% and 17% reductions in HT29 cell viability at 32 and 64 µg/mL, respectively. This low cytotoxic profile supports the peptide’s therapeutic potential, although further optimization will be essential to balance efficacy and safety. Similar studies have highlighted the importance of dose optimization to maximize antibacterial efficacy while minimizing host cell damage^18^. The peptide’s design, featuring a net positive charge of + 8.5 and a theoretical pI of 12.48, was strategically optimized for interaction with the negatively charged membranes of multidrug-resistant *A. baumannii*. Incorporation of multiple arginine (R) and lysine (K) residues enhances electrostatic binding, while hydrophobic amino acids (F, A, V, H) improve membrane insertion and stability. Such rational sequence design is a cornerstone of modern AMP engineering, where the balance between cationic charge and hydrophobicity determines both potency and selectivity^29^. Compared to natural AMPs such as Pleurocidin, a 25-amino-acid peptide with a different charge distribution^20^, the designed peptide demonstrated superior antibacterial performance against *A. baumannii*, evidenced by a MIC of 64 µg/mL and a 75% reduction in persister cell populations (*p* < 0.0001). These findings highlight how targeted structural optimization can enhance both antimicrobial and antibiofilm activities. Previous reports have also shown that engineered AMP mixtures can disrupt mature biofilms and reduce bacterial burdens in in vivo models of *A. baumannii* infection, supporting the translational potential.

In summary, the designed AMP exhibited potent antibacterial, antibiofilm, and anti-persister activities against *A. baumannii*, highlighting its promise as a next-generation therapeutic candidate against multidrug-resistant pathogens. These results emphasize the potential of AMPs to overcome conventional resistance mechanisms and complement existing antibiotic therapies. Beyond *A. baumannii*, the principles applied in this study (rational design, optimization of charge, hydrophobicity balance, and targeted disruption of resistance pathways) can be adapted to develop peptide-based agents against a broad spectrum of bacterial species. The growing global burden of antimicrobial resistance calls for innovative strategies that move beyond traditional antibiotics. By harnessing the molecular versatility of AMPs and integrating them into future treatment regimens, we may advance toward more effective and sustainable solutions for combating resistant infections and improving clinical outcomes.

The future of AMPs in clinical applications appears highly promising; however, sustained research efforts and close collaboration among scientists, clinicians, and pharmaceutical developers will be crucial to translate these findings into effective therapies. As our understanding of microbial resistance deepens and the complex interplay between host defenses and pathogens becomes clearer, insights from studies such as this will be instrumental in shaping the next generation of antimicrobial interventions.

## Limitations of the study

Despite the promising outcomes, this study has several limitations that should be acknowledged. First, all experiments were conducted in vitro using the *A. baumannii* ATCC 17,978 reference strain, which, although well-characterized, may not fully represent the diversity and resistance mechanisms of contemporary clinical isolates. Therefore, the peptide’s efficacy should be further evaluated against a broader range of multidrug-resistant clinical strains.

Second, cytotoxicity and viability assays were conducted over short-term exposure intervals (1 h, 4 h, and 24 h). We did not evaluate extended incubation periods of 48–72 h or longer, during which host-cell responses, peptide stability, or persister cell resuscitation dynamics may differ. Therefore, future work should assess longer exposure profiles to better characterize the peptide’s safety and functional persistence.

Third, while the observed upregulation of *pmr*B and *las*I genes suggests bacterial adaptive responses to peptide stress, the underlying molecular mechanisms were not investigated through proteomic or lipidomic analyses. Such analyses would help determine whether these transcriptional changes result in functional resistance or alterations in membrane composition.

Fourth, although the peptide demonstrated both antibiofilm and anti-persister effects, these assays were performed under static and simplified conditions. Biofilms can behave differently under dynamic or host-like environments, which warrants further exploration.

Finally, this study did not examine the peptide’s stability, degradation kinetics, or activity in physiological fluids (e.g., serum, plasma), factors that are essential for assessing its translational potential for in vivo or clinical applications. Future research should incorporate in vivo infection models, evaluations against diverse clinical isolates, and mechanistic analyses to validate and extend these findings.

## Conclusion

In summary, the rationally designed AMP demonstrated potent antibacterial, antibiofilm, and anti-persister activities against *Acinetobacter baumannii* ATCC 17,978. Its cationic and amphipathic structure facilitated strong interactions with bacterial membranes, leading to rapid membrane disruption and substantial inhibition of biofilm formation. The peptide also exhibited low cytotoxicity, maintaining acceptable safety toward human epithelial cells. Moreover, the observed modulation of virulence-and resistance-associated genes, including *pmr*B and *las*I, indicates that the peptide exerts a multifaceted stress response on bacterial regulatory pathways. Collectively, these findings underscore the potential of this peptide as a promising lead compound for the development of next-generation therapeutics against multidrug-resistant *A. baumannii*. Nevertheless, further investigations involving diverse clinical isolates, dynamic biofilm models, and in vivo infection systems are warranted to validate its efficacy, stability, and safety under physiologically relevant conditions.

## Materials and methods

### Preparation of the study strain

The reference strain *A. baumannii* ATCC 17,978, originally isolated from a neonatal meningitis case, was used in this study. This strain is widely employed as a model for *A. baumannii* pathogenicity and antimicrobial testing, as it shares key virulence and resistance-associated features with nosocomial isolates^30^. The bacterium was cultured from a single colony on Mueller-Hinton agar (MHA) and subsequently inoculated into 5 mL of Brain Heart Infusion (BHI) broth, followed by overnight incubation at 37 °C with shaking at 180 rpm to reach a mid-exponential growth phase.

### In silico design and evaluation of the AMP

Several peptide candidates were initially designed in silico using the AntiBP2 ^31^ and DBAASP v3 ^32^ platforms to predict antimicrobial potential and membrane-targeting efficiency. Following previously published studies^33,34^, among the generated sequences, a single 20-amino-acid linear cationic peptide (RRFFKKAAHVGKHVGKAARR) was selected based on optimal physicochemical characteristics, including high cationic charge, amphipathicity, and predicted antimicrobial activity. The amino acid composition was rationally chosen to balance electrostatic and hydrophobic interactions: arginine and lysine residues enhance electrostatic attraction to negatively charged *A. baumannii* membranes, while phenylalanine, alanine, valine, and histidine contribute to membrane penetration and stability. To estimate the physicochemical properties of the designed peptide, the ProtParam server (https://web.expasy.org/protparam/) was used to calculate key parameters, including the isoelectric point (pI), molecular weight, grand average of hydropathicity (GRAVY), net charge, number of positively and negatively charged residues, aliphatic index, instability index, and overall hydrophobicity. The PepSMI tool (https://www.novoprolabs.com/tools/convert-peptide-to-smiles-string) was employed to generate the SMILES representation of the peptide. The three-dimensional structure of the peptide was predicted using the PEP-FOLD4.0 server (https://mobyle2.rpbs.univ-paris-diderot.fr/cgi-bin/portal.py#forms::PEP-FOLD4)^35^. The helical wheel projection was constructed using the HELIQUEST tool (https://heliquest.ipmc.cnrs.fr/cgi-bin/ComputParamsV2.py)^36^. Peptide synthesis was performed by GenScript (USA) with > 95% purity. Specific primers targeting pmrB and lasI genes, key regulators of resistance and quorum sensing, were designed using NCBI Primer-BLAST, validated for specificity, and their sequences, product sizes, and melting temperatures are listed in Table 1.

**Table 1:** Sequence, product size and melting temperature of primers.

Genes	Primer sequence (5՛→ 3՛ )		PS*	Tm (°C)	References
*luxS* (*lasI*)	F	TCAAATCCGCCTTCCTCTGC	98 bp	60	In this study
	R	TTTGCGCCTTGTTCTCTTGC			
*pmrB*	F	ACGAACACCTGTGACTGCAT	112 bp	60	In this study
	R	CAAATGCTGAATACGCGCCA			
*phoP*	F	ACCGTGGGAAACTGAACCAA	115 bp	59	In this study
	R	TCCAAAGCTGGCGTAGTTGT			
16srRNA	F	CCGCCACCAAAAGTGACAAG	190 bp	60	In this study
	R	TTGGGTCGACTCCTGCTTTC			

### Determination of minimum inhibitory concentration (MIC) and minimum bactericidal concentration (MBC)

The antibacterial activity of the designed peptide against *A. baumannii* was evaluated using the broth microdilution method according to CLSI guidelines^37^. Colistin sulfate was included as a comparator because it serves as a last-resort therapeutic agent for *A. baumannii* infections and provides a clinically relevant benchmark for evaluating the peptide’s efficacy^38^. The AMP was dissolved in sterile distilled water to prepare a stock solution of 1024 µg/mL, which was stored at -20 °C and used for twofold serial dilutions. Colistin sulfate stock solution was prepared at the same concentration under identical conditions. For both agents, final concentrations in the assay ranged from 4 to 512 µg/mL.

Each well of a sterile 96-well microtiter plate received 50 µL of Mueller–Hinton broth and 50 µL of the corresponding dilution. The bacterial inoculum, adjusted to a 0.5 McFarland standard (1.5 × 10⁸ CFU/mL), was diluted 1:150 in broth, and 50 µL was added to each well. Plates were incubated at 35 °C for 24 h, and bacterial growth was quantified by measuring optical density at 600 nm (OD₆₀₀). The MIC was defined as the lowest concentration showing no visible growth^39^. To determine the MBC, 10 µL from wells showing no visible growth were subcultured onto fresh Mueller–Hinton agar plates and incubated at 35 °C for 24 h. The MBC was recorded as the lowest concentration at which no bacterial colonies were observed^40^. Positive controls consisted of *A. baumannii* grown in Mueller–Hinton broth without any antimicrobial agent, while negative controls contained only sterile broth to verify medium sterility. All MIC and MBC assays were performed in triplicate and repeated independently on three separate occasions, and the modal values were reported from these replicates.

### Anti-biofilm formation assay

The ability of the designed peptide to inhibit biofilm formation by *A. baumannii* was evaluated using a crystal violet microtiter plate assay^41^. An overnight culture of *A. baumannii* in Tryptic Soy Broth (TSB) supplemented with 1% glucose was adjusted to an optical density (OD₆₀₀) of 0.22, corresponding to the logarithmic phase. A 200 µL aliquot of the bacterial suspension was added to the wells of a sterile 96-well flat-bottom plate. The peptide was added at a final concentration of 64 µg/mL, while control wells contained no peptide. Plates were incubated statically at 37 °C for 24 h to allow biofilm development.

After incubation, planktonic cells were removed, and the wells were gently washed twice with phosphate-buffered saline (PBS, pH 7.4) to remove non-adherent bacteria. Biofilms were stained with 1% crystal violet for 20 min, washed twice with PBS, and the bound dye was solubilized with 95% ethanol. The optical density was measured at 570 nm using a microplate reader.

Biofilm inhibition was calculated as the percentage reduction in OD compared with untreated controls. All experiments were performed in triplicate and independently repeated three times for reproducibility.

### Persister cell assay

The formation and survival of persister cells were evaluated following exposure to colistin and the designed AMP, based on previously described protocols with minor modifications^42^. A single colony of *A. baumannii* ATCC 17,978 was inoculated into 5 mL of Brain Heart Infusion (BHI) broth and incubated overnight. The overnight culture was diluted in fresh BHI to an OD₆₀₀ of 0.25 (approximately 10⁸ CFU/mL).

To induce and quantify persister cells, the bacterial suspension was exposed to colistin and the designed peptide at 10× their respective MICs for 4 h at 37 °C. Untreated bacterial cultures incubated under identical conditions served as growth controls, and vehicle controls (sterile distilled water) were included where appropriate. After treatment, aliquots were collected, serially diluted in sterile PBS, and plated on Mueller–Hinton agar to determine surviving colony-forming units (CFUs). The number of surviving cells after high-dose antimicrobial exposure was considered the persister population. All assays were conducted in triplicate and repeated independently three times to ensure reproducibility.

### RNA extraction and cDNA synthesis

To assess gene expression changes, *A. baumannii* cultures were treated with the peptide at sub-MIC concentrations (32 µg/mL) for 1, 4, and 24 h. Total RNA was extracted using the High Pure RNA Isolation Kit (Roche, Germany) according to the manufacturer’s instructions, and RNA integrity was verified via agarose gel electrophoresis. Complementary DNA (cDNA) was synthesized from 1 µg RNA using the Transcriptor First Strand cDNA Synthesis Kit (Roche, Germany), following the recommended thermal cycling conditions.

### Real-time PCR analysis

Quantitative real-time PCR was performed using SYBR Green Master Mix (Roche, Germany) on a LightCycler 480 system to assess the expression of *phoP*, *pmr*B, and *lasI* genes. Each 20 µL reaction contained 10 µL SYBR Green Master Mix, 1 µL of each primer (10 µM), 2 µL of cDNA, and nuclease-free water. The 16 S rRNA gene served as an internal reference to normalize gene expression levels. Thermal cycling conditions were: initial denaturation at 95 °C for 10 min, followed by 45 cycles of 95 °C for 15 s, 60 °C for 30 s, and 72 °C for 20 s. Relative gene expression was calculated using the 2⁻ΔΔCt method^43^, and statistical significance was assessed using t-tests with a threshold of *p* < 0.05.

### Cytotoxicity and antibacterial activity assessment

Human colorectal adenocarcinoma HT29 cells (National Center for Genetic Resources, Iran) were used to evaluate the cytotoxicity of the peptide, as they are a well-established epithelial cell model for in vitro toxicity studies^44^. Cells were cultured in T75 flasks with RPMI-1640 medium supplemented with 20% fetal bovine serum (FBS), 1% sodium pyruvate, and 1% non-essential amino acids at 37 °C in a 7% CO₂ atmosphere. Cytotoxicity was assessed using the MTT assay^45^. Briefly, HT29 cells were seeded in 96-well plates and treated with the peptide at concentrations corresponding to the MIC (32 and 64 µg/mL) for 24 h. Cell viability was determined by adding MTT reagent and measuring the absorbance at 570 nm. The percentage of viable cells was calculated using the formula:

$${\mathrm{Cell}}~{\mathrm{viability}}~\left( \% \right){\text{ }} = {\text{ }}\left( {{\mathrm{OD}}_{{{\mathrm{treated}}}} /{\text{ OD}}_{{{\mathrm{control}}}} } \right){\text{ }} \times {\text{ 1}}00$$

The IC₅₀ value, representing the concentration that inhibits 50% of cell viability, was calculated from dose–response curves.

For antibacterial activity, HT29 monolayers were infected with *A. baumannii* at a multiplicity of infection (MOI) of 1:10 (2 × 10⁶ CFU/well) for 60 min at 37 °C under microaerophilic conditions. After infection, extracellular bacteria were eliminated with 100 µg/mL gentamicin for 1 h. Peptide was then added at 32 and 64 µg/mL for 4 h. Cells were subsequently lysed using 0.1% Triton X-100, and intracellular CFUs were enumerated on tryptic soy agar (TSA) after 24 h incubation. Colony counts were compared with untreated controls to assess the peptide’s antibacterial effect.

### Statistical analysis

All experiments were performed in triplicate and independently repeated at least three times. Data were presented as mean ± standard deviation (SD). Statistical analyses were conducted using GraphPad Prism version 9.0 (GraphPad Software, USA), SPSS version 26 (IBM, USA), and Microsoft Excel 2019. Differences between treated and control groups were assessed using one-way analysis of variance (ANOVA) followed by Tukey’s post hoc test for multiple comparisons. Pairwise comparisons were performed using an independent-samples t-test, where appropriate. A *p*-value < 0.05 was considered statistically significant.

## Data Availability

No datasets were generated or analysed during the current study.
